# A Novel Approach with Supra- and Retro-hepatic Cavocaval Bypass for Short Segmental Occlusion of Inferior Vena Cava in Budd-Chiari Syndrome

**DOI:** 10.4021/gr2009.08.1309

**Published:** 2009-07-20

**Authors:** Fu Tian Du, Hong Feng Lin, Wei Ding, Xiao Xia Geng, Sen Li

**Affiliations:** a Department of Hepatobiliary Surgery, Weifang People’s Hospital, Weifang 261041, China

**Keywords:** Budd-Chiari syndrome, Cavocaval bypass, Abdominal approach, Thoracolaparotomic approach, Cavoatrial bypass

## Abstract

**Background:**

Budd-Chiari syndrome (BCS) is defined as chronic, progressive and congestive liver dysfunction resulting from obstruction of the outflow of inferior vena cava (IVC) and/or hepatic veins. One of the common types of BCS is short segmental occlusion of retrohepatic IVC (SSOR-IVC) accompanied by varied extent of obstruction of intrahepatic veins. The mainstay of surgical treatment at present for SSOR-IVC is cavoartrial bypass via thoracolaparotomic approach, in which thoracic and pulmonary complications intra- and/or post-operation are common. We have developed an abdominal approach using suprahepatic and retrohepatic inferior vena cavocaval bypass to treat SSOR-IVC, herein we compared it with the conventional thoracolaparotomic approach.

**Methods:**

From 2005 to 2008, we performed suprahepatic and retrohepatic inferior vena cavocaval bypass using artificial vessel in 16 BCS patients with SSOR-IVC (group A), we compared the results of this new modality with that using traditional thoracolaparotomic approach in 18 patients (group B) from 2001 to 2004.

**Results:**

In group A, one patient had intraoperative acute cardiac failure due to rapid opening of the bypassed vessel, and the symptom was resolved immediately through prompt management, while the others were not eventful during or post-operation. The length of artificial vessel required was 6 to 8 cm, and all patients had no graft vessel thrombosis after 10 to 55 months follow-up. In group B, one patient had intraoperative acute pericardial tamponment due to anastomotic leakage. The total occurrence rate of postoperative complication was 27.8%, including three pleural effusions, one pulmonary infection and one acute pericarditis. The length of the artificial vessel required was 12 to 14 cm. Three patients had graft vessel thrombosis at 37, 42 and 58 months post-operation, respectively.

**Conclusions:**

The abdominal approach for suprahepatic and retrohepatic cavocaval bypass have advantages as follows over the traditional thoracolaparotomic approach for cavoartrial bypass: 1) Less traumatic with fewer postoperative thoracic and pulmonary complications; 2) A shorter artificial vessel required to facilitate endothelial seeding for improved long term patency; 3) Void of risk of fatal pericardial tamponment; 4) Prevention of acute pericarditis due to pericardial irritation by the artificial vessel in the thoracolaparotomic approach. We concluded that this novel abdominal approach is a safe and effective technique for treatment of SSOR-IVC.

## Introduction

Budd-Chiari syndrome (BCS) is defined as chronic, progressive and congestive liver dysfunction resulting from obstruction of the outflow of the inferior vena cava (IVC) and/or hepatic veins [1, 2]. Wang et al [3] reviewed 2564 cases of BCS in China, among them, 433 were typed with short segmental occlusion of retrohepatic IVC (SSOR-IVC). Clinically, most SSOR-IVC are accompanied by various extent of obstructive lesion in intrahepatic veins, which can be further classified into 3 pathologic types: Type 1, partial intrahepatic venous obstruction; Type 2, complete intrahepatic venous obstruction with short hepatic vein dilation; and Type 3, complete intrahepatic venous obstruction without short hepatic vein dilation [4]. Medical intervention is not effective for the BCS with SSOR-IVC. The effective treatment is surgical intervention to decrease or eliminate the hypertension of IVC and portal vein (PV) [5, 6]. Type 3 intrahepatic obstruction is usually treated with mesoatrial bypass. The mainstay of the surgery for Type1 and 2 SSOR-IVC is cavoartrial bypass via the thoracolaparotomic approach [7]. However, the thoracolaparotomic approach is commonly accompanied by thoracic and pulmonary complications intra- and/or post-operation. In 2005, we developed a novel abdominal approach for suprahepatic and retrohepatic inferior vena cavocaval bypass for the treatment of type 1 or type 2 SSOR-IVC. Since then, we have performed 16 operations with the abdominal approach. This study is to summarize the abdominal approach and compare it with the conventional thoracolaparotomic approach in the treatment of the patients with type 1 or type 2 SSOR-IVC.

## Methods

### Clinical characteristics of patients

From 2005 to 2008, 16 consecutive cases (group A) were performed suprahepatic and retrohepatic inferior vena cavocaval bypass, these patients had short segmental (< 3 cm) occlusion of IVC accompanied by type 1 or type 2 intrahepatic venous obstruction, aging from 25 to 65 years. The duration of the illness ranged from 0.5 to 32 years. Eighteen consecutive cases (group B) of same pathological type were selected for retrospective comparison, who received traditional thoracolaparotomic cavoatrial bypass from 2001 to 2004. All the patients had similar preoperative clinical symptoms and signs including upper quadrant abdominal fullness, dyspepsia, superficial varices of the tharacoabdominal wall esophageal varies and hepatosplenomegaly. The preoperative Child-Pugh scores of liver function in group A were class B (n = 11) and C (n = 5), while in group B there were 12 cases of class B and 6 cases of class C. Ascites existed in 13 cases of group A, and in 15 of group B. All class C patients in both groups were treated with liver protective drugs, diuretics, and albumin infusion. The diagnosis was confirmed by combined superior and inferior cavography and percutaneous transhepatic hepatovenography.

### Operation procedure

The abdominal approach was performed under general anesthesia. A bilateral subcostal incision was made with an upper midline extension and removal of xiphoid process to achieve excellent exposure of the right lobe of the liver. The right lobe was mobilized to the left to fully expose the right lateral wall of retrohepatic IVC which was isolated for a length of about 8 cm from hepatic vein outlet with a mandatory protection of the short hepatic veins. The sub-phrenic IVC was carefully isolated and maximized to 2.5 – 3 cm in length for later anastomosis. This may be assisted by a careful isolation of the IVC at the entrance to the diaphragm without penetration to the pericardium. A 6 – 8 cm long, 1.6 cm in diameter artificial vessel with stent spring (Gore-Tex, USA) was prepared and beveled with 40° angle at both ends for an end-to-side anastomosis. The retrohepatic IVC distal to the occlusion lesion was longitudinally clamped with a curved vascular clamp for preparation of distal anastomosis. A 1.8 cm linear venotomy immediately distal to the occlusion lesion was made and then an end-to-side anastomosis of the graft to the IVC was performed using 4-0 prolene with interrupted and mattress sutures. The proximal end-to-side anastomosis of graft to subphrenic IVC was made in the same manner. As required, a groove on the liver surface was created with a superficial incision to facilitate the alignment of the graft vessel to both anastomosis. The clamp for the distal anastomosis was released allowing blood to fill the graft vessel and a needle puncture was made on the graft vessel to discharge the air inside. The proximal anastomosis was unclamped in a very slow manner to prevent acute right heart failure due to abrupt overload to the right heart. The patency of the bypass was confirmed by intraoperative ultrasonography in all cases.

The conventional thoracolaparotomic approach of cavoatrial bypass for SSOR-IVC adopted in this study was described previously by Sun et al [8]. Briefly the thoracolaparotomy was performed through a 7th-8th intercostal incision to gain access to the retroheatic region. A 12-14 cm long, 1.6 cm in diameter artificial vessel with stent spring (Gore-Tex, USA) was used for a bypass between the retrohepatic IVC distal to the occlusion lesion and the right atrium.

## Results

Retrospective comparison of the abdominal and thoracolaparotomic approaches is illustrated in Table 1. In group A (n = 16), the mean operation time was 250 ± 28 min versus 340 ± 36 min in group B, Mean ± STDV, P < 0.05. Postoperative ICU stay was 2.8 ± 0.3 days in group A versus 5.2 ± 0.8 days in group B, Mean ± STDV, P < 0.05. All operations in group A were uneventful except one patient suffering from intraoperative acute cardiac failure due to rapid opening of the bypass which was resolved immediately through prompt management. In group A, no postoperative complication or graft vessel thrombosis was found after 10 to 55 months follow-up. In group B, one patient had intraoperative acute pericardial tamponment due to anastomotic leakage, the occurrence rate of postoperative complications was 27.8%, with three cases of pleural effusion, one pulmonary infection and one acute pericarditis, three patients had graft vessel thrombosis at 37, 42 and 58 months post-operation respectively.

**Table 1:** Comparison of abdominal approach and conventional thoracic approach

Clinical data	Abdominal approach	Thoracic approach
Operation Time (min)	250 ± 28	340 ± 36
		
ICU stay(days)	2.8 ± 0.3	5.2 ± 0.8
		
Intra-op complications	acute right heart failure, n = 1	acute pericardial tamponment, n = 1
		
Post-op complications	0	27.80%
		pleural effusion, n = 3
		pulmonary infection, n = 1
		pericarditis, n = 1
		
Post-op thrombosis	0	3
		

## Discussion

The first case of Budd-Chiari syndrome (BCS) was reported by George Budd in 1845, and then in 1899 Hans Chiari first reported the findings of occlusion of intrahepatic vein in BCS. The incidence is higher in the Eastern countries and South Africa with the lesions predominantly at the retrohepatic portion of IVC, while in Western countries the lesion occurred mainly in the intrahepatic veins [9-11].

Budd-Chiari syndrome is characterized with portal and IVC hyertension due to obstruction of the main hepatic veins or/and supra- or retrohepatic IVC. One of the common lesions in BCS, especially among Eastern countries, is the short segmental occlusion of retrohepatic IVC (SSOR-IVC) accompanied by various obstruction of hepatic veins [12-13]. According to a recent operation review in 2564 BCS cases in China, 433 were reported to have the SSOR-IVC and treated with cavoatrial bypass [3]. The conventional surgical intervention on SSOR-IVC is cavoartrial bypass via thoracolaparotomy, and it was indicated only for type 1 and type 2 SSOR-IVC [14-17]. This thoracolaparotomic approach offers a better exposure for anastomosis of graft vessel to the IVC and the atrium, but is accompanied by postoperative complications due to the operative trauma in thoracolaparotomy [18-20]. The postoperative pericarditis may be caused by the irritation of the artificial graft [21]. In our previous thoracolaparotomic approach operations, the postoperative complication occurred in 27.8% cases. The thoracolaparotomic approach also has a reduced patency rate at long-term due to application of a longer artificial vessel (12 - 14cm). The length of the artificial graft is critical for prevention of thrombosis in the bypass because longer artifical vessel may impede the cellular seeding to form an intact endothelial layer in the graft vessel [22-24]. An experimental study found that only 4 to 5 cm of the artificial vessel to the anastomosis can be completely covered by the endothelial cells 12 weeks after operation [25-29]. According to a recent review, the patency rate of cavoatrial bypass is 90.7% at one year after operation, and reduced to 77.1% at 3 years, 61.1% at 5 years, and 50% at 10 years [25-29].

We have developed an abdominal approach for SSOR-IVC with partial hepatic venous obstruction or complete obstruction with dilation of short hepatic vein. This procedure of suprahepatic and retrohepatic cacocaval bypass has the following advantages over the conventional thoracolaparotomic approach of cavoartrial bypass: 1), the operation is less traumatic with fewer postoperative complications; 2), Shorter artificial vessel (6 – 8 cm) is needed, this facilitates endothelial seeding to improve long term patency; 3), Void of the risk of fatal pericardial tamponment; 4), Prevention of acute pericarditis caused by pericardial irritation by the artificial vessel in the thoracolaparotomic approach. Thus, our comments and experiences on the abdominal approach include: only 2.5 - 3.0 cm of subphrenic IVC is required for the anastomosis, and it can be achieved with isolation at its entrance to diaphragm with special care for not penetrating the pericardium; a groove incision on the liver may assist the alignment of the graft vessel to both anastomoses, unclamping the proximal anastomosis is always in a very slow manner to prevent acute right heart failure.

In conclusion, our preliminary results showed that, compared with the traditional thoracolaparoctomic approach, this novel abdominal approach is a safe and effective procedure for treatment of short segmental occlusion of inferior vena cava in Budd-Chiari syndrome with improved long term patency and reduced postoperative complications.
